# Stent-Over-Sponge (SOS) as a Rescue Technique for Leak Post-Bariatric Surgery: Experience From Hôpital du Sacré-Coeur, Canada

**DOI:** 10.7759/cureus.77285

**Published:** 2025-01-11

**Authors:** Majed Alanazi, Bandar Ali, Ibrahim Alonazi, Pierre Y Garneau, Denis Ronald, Radu Pescarus

**Affiliations:** 1 Surgical Gastroenterology, Minimally Invasive and Bariatric Surgery, Hôpital du Sacré-Coeur, Centre Intégré Universitaire de Santé et de Services Sociaux, Montréal, CAN

**Keywords:** bariatric surgery, laparoscopic surgery, leak, sponge, stent

## Abstract

Leaks and fistulas are serious complications following gastrointestinal surgeries, traditionally managed by self-expandable metal stents and endoscopic vacuum therapy. The stent-over-sponge (SOS) technique is a new modality used as a rescue option when other interventions fail.

This report presents the case of a 60-year-old female patient who underwent revisional bariatric surgery and developed a leak post-operation. Initial management included endoscopic debridement and the placement of an Endo-VAC system. Due to technical difficulties, the sponge was left in an endoluminal position, leading to migration. A partially covered stent was placed to prevent further migration and facilitate healing. The patient experienced complications, including hematemesis, but ultimately achieved complete leak closure and is asymptomatic six months post-treatment.

In this case, the SOS technique demonstrates its safety and efficacy in dealing with post-operative leaks in patients having undergone bariatric surgery, which would justify performing more extended evaluative studies.

## Introduction

A gastrointestinal (GI) leak or fistula occurs when the continuity of the gastrointestinal tract (GIT) wall is disrupted [1]. Leaks and fistulas are fearsome complications following GIT surgeries that affect morbidity and mortality [2]. Causes can be either spontaneous, due to underlying GI pathology, or iatrogenic [3]. Etiologies can also be classified as mechanical and ischemic [4].

There are variable clinical signs of a leak. Some may be subclinical, and others manifest as pleural collections or sepsis. A high level of suspicion cannot be overemphasized. Diagnosis depends on clinical signs, laboratory tests, and radiological and endoscopic investigations [5].

Management depends on the severity of anastomotic necrosis and leakage. In all cases, conservative measures should be initiated: nasogastric tube (NGT) suctioning, parenteral feeding, intravenous antibiotics, proton pump inhibitors, and intravenous anticholinergics. In the past, leaks were managed surgically. Nowadays, endoscopic measures have become the gold standard [6]. Self-expandable metal stents (SEMSs) are an established method of endoscopic intervention for leak management [7]. Furthermore, endoscopic vacuum therapy (EVT) is also a new promising alternative [8]. No evidence suggests one of the two as a superior option [9].

Stent-over-sponge (SOS) is a new modality used as a rescue option in case other interventions fail. It was done in the Department of Minimally Invasive and Bariatric Surgery, Québec Montréal, Canada. The literature describes it as a good option for treating leaks after upper GI oncological resection. We reported a revisional bariatric surgery complicated by the leak, during which SOS was used as a rescue intervention. To our knowledge, there are no other case reports of SOS in revisional bariatric surgery so far. 

## Case presentation

**Figure 1:**
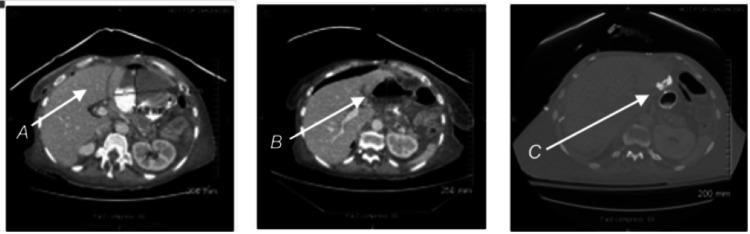
Multiple follow-up CT scans: A: Free contrast outside the lumen. B: Abscess formation. C: Endo-VAC and stent in place Arrow A indicates the area of free contrast outside the lumen, highlighting the site of the leak; Arrow B points to the formation of an abscess, showing the accumulation of fluid in the abdominal cavity; Arrow C marks the position of the Endo-VAC and stent in place, demonstrating the intervention used to manage the leak.

A 60-year-old woman presented with a history of gastric banding and subsequent sleeve gastrectomies and then roux-en-Y gastric bypass. She underwent bowel resection with extended adhesiolysis via laparotomy. The patient underwent many bariatric surgeries, starting with the gastric band, then the band was removed, and then she underwent sleeve gastrectomy. After this, she developed reflux symptoms, for which she underwent roux and gastric bypass, all previous surgeries by laparoscopy. Then she developed a small bowel fistula which was explored and a lot of adhesions were found, which were converted to laparotomy. On post-operative day 4, the patient became septic. An abdominal computed tomography (CT) scan (Figure 1) showed a large contrast leak near the gastro-jejunal anastomosis, with multiple intra-abdominal abscesses, for which four pigtails were inserted.

**Figure 2:**
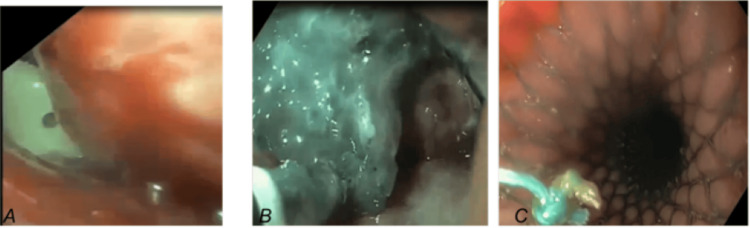
Photos from the upper endoscopy: Jackson-Pratt intrabdominal drain is seen by endoscopy, Endo-VAC insertion, and partially covered stent in place (A) Intraperitoneal abdominal drain seen while doing endoscopy; (B) Cavity was reached by endoscopy and the pus was evacuated and the sponge was applied; (C) Placing the stent

Diagnostic upper endoscopy (Figure 2) was performed under general anesthesia; it revealed complete dehiscence of the terminal staple line of the candy cane. An initial endoscopic debridement of the cavity was performed. Given the location and the large size of the leak, an Endo-VAC system on a 16F NGT was inserted. Due to angulation issues, an intra-cavitary placement was impossible, and the sponge was left within the Candy cane in an endoluminal position. Migration of the sponge into the alimentary limb was noted following two Endo-VAC changes. Therefore, a partially covered esophageal stent (WallFlexTM Boston Scientific, 23mm*155mm) was placed with its distal end on the shared wall between the Candy cane and the alimentary limb to keep the Endo-VAC from migrating. Percutaneous drainage with four pigtails of the multiple intra-abdominal collections was performed with the endoscopic treatment.

Stent and Endo-VAC removal were performed urgently 10 days after stent placement, as she developed significant hematemesis due to the patient attempting to remove the Endo-VAC herself. Complete leak closure was noted during the endoscopic control with clips of a small jejunal artery. The patient is currently asymptomatic six months after the leak resolution, with complete resolution of the abdominal accesses.

## Discussion

A GI leak or fistula occurs when the continuity of the GIT wall is disrupted. An anastomotic leak is a full-thickness GI defect involving the esophagus, anastomosis, staple line, or conduit, irrespective of presentation or identification method [10]. Causes can be spontaneous due to underlying GI pathologies such as malignancy, Crohn's disease, Boerhaave's syndrome, and tuberculosis. It can also happen iatrogenically following endoscopic, bariatric, colorectal, and oncological procedures [10]. 

Baker et al. divided causes into "mechanical-tissular" and "ischemic" causes. Technical or "mechanical-tissular" causes are defined as those due to the surgery itself, including direct injury to tissue or misfiring during stapler usage. These usually appear early within the first two days after surgery. In contrast, "ischemic causes" commonly become evident around 5-6 days postoperatively [11].

There are variable clinical signs of a leak; some may be subclinical, and others can manifest as pleural collections or sepsis. A gold standard for diagnosing leaks has yet to be established [12]. Thus, a high level of suspicion must be addressed. Whenever a complication is suspected after GI surgery, diagnostic tests should be carried out immediately, as any delay in management initiation can affect the patient's prognosis. A leak can manifest as fever, leukocytosis, or surgical site infection. An abnormal heart rate, often manifested as atrial fibrillation, may be the first sign of a leak [13]. Inflammatory marker levels (CRP and ESR) are elevated 3-4 days post-operatively and can detect leaks [14]. Fluid analysis of the drain and saliva or gastric content are apparent signs of a leak. Thus, analysis of drain fluid for amylase levels can detect a leak as soon as post-operative day 4 and be more accurate than a barium esophagogram [15]. CT is the best non-invasive diagnostic method for gastric leak detection and confirmation [16-19]. The early post-operative gastrograffin swallow test and upper GI endoscopy are described tests for leak detections post-surgery.

Partially or fully covered stent insertion successfully manages leaks [20,21]. Additionally, other endoscopic techniques such as endo-clips [22], fibrin glue [23], and the new Scope Clip (OTSC) [24] can treat some leaks successfully. Some lesions, however, can remain for weeks or months despite various efforts.

Bartella et al. published a case report in 2019 about SOS as a rescue option in patients with complex post-operative anastomotic leaks after esophagectomy for adenocarcinoma of the distal esophagus [25]. Endoscopy diagnosed the leak on post-operative day 8. In the beginning, EVT was used alone. After seven days of EVT, there was no improvement. Thus, SOS was employed for eight days. Improvement was noticed with the aid of a CT scan. The SOS system was removed, and SEMSs were deployed for a few weeks. Endoscopy confirmed complete healing on the 46th day. The course was complicated with aspiration pneumonia.

We report here our experience with SOS in the management of leaks post-revisional bariatric surgery. This case report is the first to use SOS to manage leakage in bariatric surgery to be reported in the literature. 

In our case, the patient developed leakage after the surgery on day 4, which CT confirmed. Initial debridement of the anastomosis was done, and the Endo-VAC system was deployed. There was technical difficulty due to angulation issues related to the anastomosis type. Thus, putting the sponge in the cavity of the leakage defect was impossible, and the sponge was put endoluminal. Nonetheless, sponge migration was detected by follow-up endoscopy. So, the SOS system was assembled using a partially covered stent with an Endo-VAC system. The SOS system prevented the migration of sponges, and the healing process improved. Also, there were intra-abdominal collections for which four pigtails were inserted for drainage. The further course was complicated by hematemesis due to jejunal artery bleeding because the patient pulled on the NGT tube. Bleeding was controlled by clips applied to the artery during endoscopy. 

Previous reports described SOS in managing leaks in upper GI oncological resection [26]. We used SOS as a rescue option to manage revisional bariatric surgery. As reported in previous cases, we did not experience stenosis or aspiration pneumonia. However, hematemesis was encountered due to patient noncompliance.

## Conclusions

Leaks and fistulas are fearsome complications following GI surgeries. They have traditionally been managed by SEMSs and EVT. In this case, the SOS technique demonstrates its safety and efficacy in dealing with post-operative leaks in patients having undergone bariatric surgery, which would justify performing more extended evaluative studies. Combining the two methods is a promising technique. SOS is a new modality used as a rescue option. There are only a few subsequent reports of similar cases. We report the first case of revisional bariatric surgery, complicated by a leak, where SOS was used as a rescue intervention. It demonstrates the safety of this method. Further studies are needed to investigate this further.
